# Alpha-Synuclein—Nanoparticle Interactions: Understanding, Controlling and Exploiting Conformational Plasticity

**DOI:** 10.3390/molecules25235625

**Published:** 2020-11-29

**Authors:** Mariapina D’Onofrio, Francesca Munari, Michael Assfalg

**Affiliations:** Department of Biotechnology, University of Verona, 37134 Verona, Italy; mariapina.donofrio@univr.it (M.D.); francesca.munari@univr.it (F.M.)

**Keywords:** alpha-synuclein, amyloid fibrils, conformational flexibility, protein adsorption, protein aggregation, nano-bio interface, nanocomposite, nanoparticles, supramolecular assembly

## Abstract

Alpha-synuclein (αS) is an extensively studied protein due to its involvement in a group of neurodegenerative disorders, including Parkinson′s disease, and its documented ability to undergo aberrant self-aggregation resulting in the formation of amyloid-like fibrils. In dilute solution, the protein is intrinsically disordered but can adopt multiple alternative conformations under given conditions, such as upon adsorption to nanoscale surfaces. The study of αS-nanoparticle interactions allows us to better understand the behavior of the protein and provides the basis for developing systems capable of mitigating the formation of toxic aggregates as well as for designing hybrid nanomaterials with novel functionalities for applications in various research areas. In this review, we summarize current progress on αS-nanoparticle interactions with an emphasis on the conformational plasticity of the biomolecule.

## 1. Introduction

Alpha-synuclein (αS) is a paradigmatic and one of the most extensively investigated intrinsically disordered proteins (IDPs) [1,2]. It is an abundant neuronal protein which localizes predominantly to presynaptic terminals and binds to small synaptic vesicles [3]. The biological function of the protein remains enigmatic, although increasing evidence supports its participation in neurotransmission and synaptic plasticity, including roles in synaptic vesicle recycling and neurotransmitter synthesis and release [4,5,6]. αS has also been reported to interact with and affect a variety of proteins [7]. Soon after its discovery, the protein became infamous for being strongly linked, genetically and pathologically, to Parkinson’s disease (PD) and other neurodegenerative diseases characterized by abnormal accumulation of insoluble αS deposits [8,9,10].

Encoded by the SNCA gene, αS is a 14-kDa polypeptide which can be broadly divided into three domains: an amphiphilic N-terminal region (residues 1−60) that contains four imperfect eleven-residue amino acid repeats, a hydrophobic, amyloidogenic domain (residues 61−95, referred to as non-amyloid-β component, NAC, domain) with three additional repeats, and an acidic C terminus (residues 96−140) [9,11]. In dilute solution, αS is unstructured and best described as a dynamic ensemble of interconverting conformations [12,13,14]. Mounting evidence indicates that native oligomers exist in cells, which exhibit greater aggregation resistance than the disordered monomeric species [15,16,17,18]. Environmental changes, binding events and other stimuli may promote the transition from the soluble monomeric and small oligomeric states to higher-level oligomers, fibrils (highly ordered supramolecular nanostructures) and amorphous aggregates [12,15,19,20]. The high protein solubility and the possibility to trigger conformational changes in vitro upon exposure to specific environments and stimuli, have made αS a popular model for structural and aggregation studies of IDPs [1,21].

In the context of amyloidogenic, unfolded peptides and proteins like αS, nanoparticles (NPs) have attracted interest as artificial receptors or chaperones against the formation of toxic aggregates [22]. NP surfaces, acting as a scaffold for protein adsorption, could provide the means to redirect aggregation pathways, sequester/correct misfolded structures, retard or accelerate aggregation and disaggregate assemblies. NPs are versatile platforms, they can be prepared in a wide range of sizes and with diverse surface chemistries. In principle, by careful design of the NPs it should be possible to control their interactions with biological components and develop artificial receptors capable of biomolecular recognition [23,24,25,26,27,28]. Among the vast array of potential applications that rely on optimized target recognition, NPs represent a promising alternative to conventional small drugs for targeting protein-protein interactions associated with pathological conditions [24] and indeed have emerged as a new class of therapeutics [29].

The nanometer scale of their size makes NPs able to interact with cellular systems and biomolecular networks and to reach targets of biomedical interest [30]. Upon exposure to biological media, NPs tend to be covered by a layer of biomolecules, generally consisting of an internal, long-lived layer (termed hard corona) and a more loosely associated, external layer (termed soft corona) [30]. The protein corona mediates the interactions with the living systems and determines the physiological responses [30,31]. Unintended protein adsorption onto NPs may perturb the protein’s activity as a consequence of binding-induced changes in structure, stability or the exposure of recognition sites [32]. It is thus essential to characterize both the structural and dynamic organization of the corona and the modes of binding of distinct biomolecules to the NP surface [33].

The ability of ad-hoc prepared NPs to target and associate to specific proteins can be extended to the development of novel hybrid materials composed of proteins and NPs, which feature unique attributes not attainable with the separate components [34]. Protein-NP bioconjugation is an attractive method to fabricate functional materials enabling applications in sensing, delivery and other nanotechnological areas [34]. Protein molecules can be used to coat the NPs and protect them from the medium, to join multiple NPs in order to form higher order supramolecular assemblies, they can be introduced into hollow structures of the NPs or be surrounded by them [35]. The variety of conformational states of certain disordered and self-aggregating proteins may be exploited to tune the properties of the hybrid material to different purposes.

A number of groups have strived to elucidate the modes of binding of αS to simple NPs made by different core materials, including silica, gold and lipids, which exhibit biocompatibility and can be easily functionalized [36,37,38]. A variety of experimental and computational techniques were applied to gain information at the molecular and sub-molecular level on the organization of αS molecules within a hard corona, on the NP-induced structural transitions, the determinants of binding and the dynamic exchange processes at the nano-bio interface. The possibility to perturb the aggregation behavior of αS by use of NPs has attracted considerable interest [39,40]. Research efforts were largely focused on the observation of aggregation kinetics curves of αS in the presence or absence of NPs, providing insight into the aggregation process at the macroscopic level [41]. A mechanistic description of the effects of NPs on the aggregation kinetics has lagged behind, however few reports have provided preliminary insight into the microscopic events and structural conversions taking place during αS aggregation [42,43,44]. Interestingly, certain NPs were shown to interact with and disassemble preformed amyloid fibrils [40]. Tailored interactions between αS and NPs were further explored to develop reactive agents and nanobiocomposites featuring novel attributes for application in nanotherapeutics, nanooptics, nanoelectronics and other areas [45,46]. A large number of studies have been carried out to explore how NPs bind to and influence the aggregation propensity of diverse amyloidogenic proteins and peptides other than αS [22], however αS appears to have attracted greater attention than other IDPs for developing composite nanomaterials, presumably due to its unique favorable properties, such as molecular size, solubility, stability and ease of production.

The objective of this review is to summarize progress made in the study of αS interactions with NPs, with an emphasis on the conformational plasticity of the protein and its self-assembling propensity. The review is divided into three sections discussing: (1) fundamental aspects and molecular determinants of αS adsorption onto NP surfaces; (2) efforts aimed at controlling αS self-aggregation, formation of toxic assemblies and disaggregation of insoluble fibrils; (3) achievements towards the fabrication of αS-based hybrid materials presenting novel functionalities. We aim to provide the basis for better understanding the conformational properties of αS at the interface with NPs and illustrate how this knowledge may support our ability to control αS structural transitions and to design functional nanocomposites. Among the large variety of known NPs, we decided to focus our survey on the simplest type, namely particles of near-spherical shape. The selected case studies comprise both inorganic and organic materials as well as lipid nanovesicles. While the former types are attractive tools for exploratory purposes and applications, the latter are included for their great relevance as biomembrane mimics to probe αS conformational versatility and membrane surface-induced structural transitions.

## 2. Adsorption of Monomeric Alpha-Synuclein onto Nanoparticles

### 2.1. Silica Nanoparticles

The distinct physicochemical environment of the hard and the soft corona is expected to influence differently the conformational preferences of protein molecules. Recent work by Grandori and coworkers focused on the characterization of the conformations of αS and other proteins in the hard corona of silica NPs (SNPs) [37]. To prepare corona-coated SNPs, SNPs (~50 nm) were incubated with excess αS and then subjected to centrifugation and washing cycles. Based on transmission electron microscopy (TEM) analysis, the αS corona was found to be formed by a monolayer of tightly bound, collapsed molecules. Circular dichroism (CD) and Fourier-transform infrared (FTIR) spectra suggested the formation of helical segments on adsorption of αS to SNPs, however the effect was rather limited, indicating that the disordered state remained prevalent. Despite the limitations of the single experimental techniques in the analysis of protein-NP hybrids, such as possible scattering effects affecting CD experiments, the work demonstrated that a combination of them could provide useful structural insights into adsorbed protein layers.

**Figure 1:**
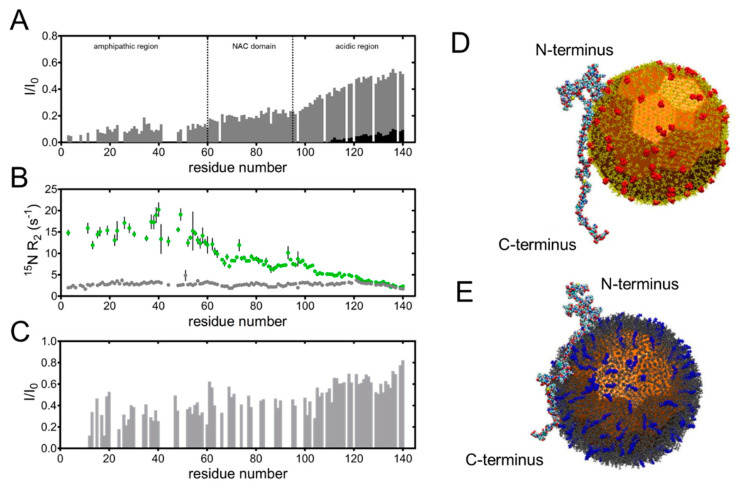
Orientation of αS molecules on the surface of nanoparticles. (**A**–**C**) Site-resolved nuclear magnetic resonance (NMR) interaction profiles revealing amino acid residues of αS involved in binding to silica nanoparticles (SNPs) on the basis of intensity attenuations or increased nuclear spin relaxation rates. (**A**) Relative signal intensities obtained from heteronuclear single quantum correlation (HSQC) spectra measured on ^15^N-enriched αS dissolved in buffer solution, in the absence of SNPs (*I*_0_) or in their presence (*I*), at two different concentrations (gray and black bars). (**B**) ^15^N transverse relaxation rate values measured in the absence (gray dots) and presence of SNPs (green dots). (**C**) Relative HSQC signal intensities measured on ^15^N-enriched αS in human blood serum containing (*I*) and not containing (*I*_0_) SNPs. Adapted from ref. [38], Copyright 2020, with permission from Elsevier. (**D**,**E**) Rendering of simulated interaction of αS with AuNPs showing the reversed orientation of the protein depending on the capping ligand. (**D**) Interaction with citrate-capped AuNPs. (**E**) Interaction with MTAB-capped AuNPs. Citrate and MTAB ligands are charged (highlighted in red and blue, respectively). Adapted with permission from Lin et al. J. Phys. Chem. C Nanomater. Interfaces. 2015, 119(36), 21035–21043. Copyright 2015 American Chemical Society.

To complement the knowledge acquired on the hard corona, a subsequent study was aimed at the characterization of αS molecules in dynamic exchange with the surface of SNPs [38]. Tira et al. used nuclear magnetic resonance (NMR) spectroscopy to gain insight into the adsorption mechanism at single-residue resolution. The direct observation of NMR signals from NP-bound proteins is generally unfeasible due to excessive line-broadening caused by the slow rotational tumbling of the hybrid species, however perturbations may be detected as intensity losses or exchange-averaged observables [47,48]. Indeed, heteronuclear single quantum correlation (HSQC) spectra and relaxation rate measurements performed on samples containing αS and SNPs revealed that the amphipathic amino-terminal domain was the primary contact with the NP surface, while the carboxy-terminal domain retained significant mobility (Figure 1A,B). An oxidized form of αS, containing four methionine sulfoxides, associated with oxidative stress, was found to exhibit similar binding properties as the unmodified species, indicating that the increased hydrophilicity did not influence the binding to SNPs. Interestingly, αS interacted with the surface of SNPs also in the molecularly crowded environment of blood serum with similar orientation as in simple buffer (Figure 1C). Additional competition binding experiments, supported by sodium dodecyl sulfate-polyacrylamide gel electrophoresis (SDS-PAGE) analysis, showed that αS was able to displace serum albumin and other proteins from the surface of NPs, while the highly basic four-repeat domain of Tau, an amyloidogenic IDP, displayed stronger affinity to SNPs, compared to αS. Thus, αS adsorption was described as a dynamic process wherein molecular exchange on the surface determines the composition and organization of the protein corona.

The capability of nuclear magnetic resonance (NMR) spectroscopy to provide atomic-resolution information on protein-NP interactions and the qualitative observation that the binding of IDPs to certain NPs is related to the polypeptide sequence, stimulated Brüschweiler and coworkers to attempt a quantitative analysis of residue-specific NMR data [49]. The authors developed an interaction model based on a quantitative NP affinity scale determined from measurements on single amino acid types. In conditions of rapid exchange between free and surface-bound states, the difference in residue-specific ^15^N-spin transverse relaxation rate constants, ^15^N-*R*_2_, observed in the presence and absence of NPs, termed Δ*R*_2_, provides a direct measure of the interaction strength between the NP and residues in the polypeptide chain. After a number of refinements, the authors came up with a binding model capable to accurately predict residue-specific binding affinities of αS and other IDPs to SNPs [50,51]. This work provided mechanistic insight into the binding of αS and SNPs, explaining the observed interaction profile in terms of the non-uniform distribution of charged and neutral amino acids as well as in terms of global and local cooperativity effects.

Overall, the binding of αS to SNPs appears as a simple reversible two-state binding mechanism mediated in large part by the lysine-rich N-terminal domain, which experiences attractive electrostatic interactions with the negatively charged, deprotonated silanol groups at the SNP surface. However, this simplified picture does not explain all observations, such as the apparently distinct involvement of the C-terminal domain at different protein/NP ratios and the formation of a slowly desorbing hard corona [38]. Furthermore, the role of intermolecular interactions in the structural organization of the protein corona have remained elusive. Diverse techniques have served to provide detailed descriptions of the organization of folded protein molecules bound to NPs [52,53,54], however similar results were not obtained for αS, likely due to the greater difficulty of obtaining clear data for an unstructured polypeptide. All of these aspects deserve attention in future studies.

### 2.2. Gold Nanoparticles

The study of the adsorption of proteins to AuNPs has invariably involved the use of capped or functionalized AuNPs. In an early work, Murphy and coworkers investigated the interaction of αS with citrate-capped 20 nm and 90 nm AuNPs [55]. The authors used dynamic light scattering (DLS) to monitor changes in the mean hydrodynamic diameter after mixing the protein with the NPs. They observed the formation of a relatively thick, strongly bound adlayer (hard corona) and a less thick, labile soft corona. An overall apparent binding constant of (2.0 ± 0.4) × 10^7^ M^−1^ was estimated from the DLS data using 20 nm AuNPs and a similar result was obtained from the analysis of plasmon band maxima in the UV−vis spectra. Fluorescence quenching was exploited to separately quantify the binding constants for the hard and the soft corona, yielding affinity constant values in the order of 10^7^ M^−1^ and 10^−3^ M^−1^, respectively. The latter value suggested that the binding of the soft corona was thermodynamically unfavorable and kinetically driven. The authors further attempted a structural analysis of NP-bound protein by CD using a stacked double-cuvette method but realized that high absorbance by the metallic core could compromise the quality of the obtained results. Therefore, they resorted to use enzymatic digestion followed by mass spectrometry (MS) analysis to gain insight into the structure of αS in the hard corona. A comparison of trypsin digestion patterns of free αS with bound αS on 20 nm citrate-capped AuNPs suggested that the protein maintained its native unstructured state when bound on AuNPs, with the N-terminal section strongly adsorbed onto the NP surface.

As opposed to citrate-capped AuNPs, which expose a negatively charged surface, poly (allylamine hydrochloride) (PAH) coated AuNPs display a positively charged surface. Murphy’s group used a similar methodology as that used with citrate AuNP to explore the structure of αS bound to PAH AuNPs [36]. The protein was found to adsorb as multilayers when present at low protein/NP ratios and eventually formed agglomerates at higher ratios. The latter condition was attendant with an increase in β-sheet structure and decrease in α-helical content, possibly explaining the tendency to form agglomerates. Apparently, the mode of adsorption could elicit the seeding of a global conformational change of αS in the sample. Based on trypsin digestion data, the protein molecules were found to adopt random orientation in the multilayered corona.

Solution NMR spectroscopy was used in a subsequent study to obtain definitive insight into the orientation of αS on both anionic and cationic AuNPs [56]. For cationic particles, in spite of using PAH, AuNPs were capped with (16-mercaptohexadecyl) trimethylammonium bromide (MTAB), a ligand that did not promote protein aggregation and therefore allowed better interpretation of the NMR data. As expected, portions of the protein that bound to NPs exhibited larger linewidths and attenuated signals. A comparison of the residue-by-residue intensity profiles collected with the anionic and cationic NPs clearly showed the reverse orientation of the protein, with the prevalently basic N-terminus acting as the anchor to citrate-capped AuNPs and the acidic C-terminus being prevalently bound to MTAB AuNPs. Besides identifying the sections in direct contact with the NPs’ surfaces, the NMR data also revealed that the unanchored portions experienced restricted motion due to their tethered condition. In both cases, the protein remained disordered upon binding to the NPs. Molecular dynamics (MD) simulations supported the observed reversal of protein binding orientation (Figure 1D,E) and additionally indicated that the central hydrophobic segment, the NAC domain, was attracted to both types of AuNPs. These results demonstrated the possibility to obtain molecular control of protein display on engineered NPs.

### 2.3. Lipid Nanovesicles

In cells, αS partitions between disordered or partly ordered cytosolic forms and phospholipid-bound states [57,58,59]. The association of αS with phospholipid membranes was linked to a role of the protein in the regulation of reserve pools of synaptic vesicles and dopamine homeostasis [6,60]. Such crucial function has spurred the investigation of αS binding to lipid nanovesicles as membrane mimics. The N-terminal 11-mer repeat sequence of αS, resembling the sequence motifs found in apolipoprotein A-I, suggested that it could form amphipathic helical lipid-binding domains [61]. Indeed, several in vitro studies have established that αS undergoes a coil-to-helix transition when binding to vesicles made by acidic phospholipids or anionic detergents [61,62,63].

In studies of αS-lipid interactions, a large variety of lipid/detergent vesicles of different composition and size have been used and several distinct experimental conditions were tested, which may in part account for the fact that results were sometimes seemingly contradictory [2,64]. The increase in helical content on binding of αS to anionic phospholipid vesicles was initially observed by CD spectroscopy and the interaction was proposed to be mediated by four N-terminal helices (region 1–60) [61]. In a subsequent study, Eliezer and coworkers used small (~5 nm) SDS detergent micelles as membrane mimics in place of larger phospholipid vesicles to facilitate observation of the lipid-bound state by NMR spectroscopy [12]. They found that binding of α-synuclein to SDS micelles elicited formation of an extended α-helix encompassing residues 1–100, while the C-terminal portion of the protein remained unassociated. Later investigations, based on NMR and partial tryptic digestion, identified a short break within the extended helical region [63,65]. Furthermore, spin probe-induced broadening of NMR signals, ^15^N relaxation measurements and fluorescence spectroscopy data indicated the presence of two N-terminal helices, positioned on the surface of the SDS micelle and separated by a flexible stretch [66]. The region of residues 61–95 was found to adopt a helical conformation but it was proposed to be partially embedded in the micelle [66]. Finally, a high resolution structural determination established that micelle-bound αS forms two curved α-helices within the N-terminal domain, connected by an ordered, extended linker in an anti-parallel arrangement, followed by another short extended region and a largely disordered tail (residues 98–140) [67].

**Figure 2:**
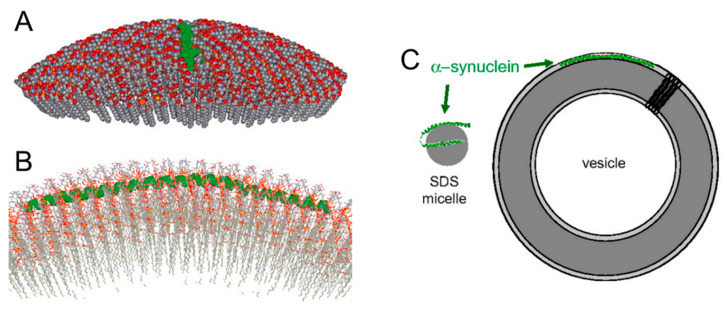
Interaction of αS with curved lipid surfaces. (**A**) Space-filled model of α-synuclein (shown in green) binding to the surface of a lipid vesicle 300 A in diameter; ≈ 25% of the outer leaflet of the vesicle is shown. The vesicle was fitted around one of the structures derived from the experimentally restrained simulated annealing—molecular dynamics (SAMD) calculations. (**B**) A closer cross-sectional view of the αS interaction with the lipid surface, with rotation through 90° from the image in A. The protein (green) follows the curved surface of the vesicle, with the helical axis positioned just below the level of the phosphate groups of the lipids. This position of the protein emerged from the SAMD calculations and reflects the immersion depths obtained from the continuous-wave electron paramagnetic resonance (EPR) data. (**C**) Cartoon representations of the structures of α-synuclein on micelles and small unilamellar vesicles (SUVs). The small and highly curved micelles cannot accommodate the extended helical structure present on the membrane. Adapted with permission from Jao et al., Proc. Natl. Acad. Sci. USA, 2008, 105 (50) 19666–19671. Copyright 2008 National Academy of Sciences.

SDS micelles have provided an invaluable system to study structural properties of lipid-bound αS, however both the chemical composition and the size do not entirely recapitulate the features of the ~50 nm presynaptic phospholipid vesicles. Thus, several studies have been conducted using either small or large unilamellar vesicles (SUVs or LUVs). A structural model of SUV-bound αS was obtained by applying simulated annealing MD restrained by the immersion depths and long-range distances obtained from continuous-wave and pulsed electron paramagnetic resonance (EPR) data [68] (Figure 2). The bound form was described as an extended helix (ca. 90 amino acids long) with a curved arrangement that follows the curvature of the vesicle surface and allows lysine residues to interact with phosphate groups, acidic residues to approach the choline groups and hydrophobic residues to associate with the lipidic moieties. Further experimental evidence, based on single molecule Förster resonance energy transfer and on the use of 100 nm LUVs, supported the extended helix model [69], however other authors concluded that the broken-helix arrangement best described the SUV-bound protein state [70]. Later studies provided evidence for coexisting populations of broken and extended helices, in part reconciling the divergent models [71,72]. It was found that relative protein/lipid concentrations and vesicle size could modulate the preference of αS for distinct helical arrangements. A multiplicity of coexisting binding modes were further proposed by Bax and coworkers, featuring slow exchange kinetics and involvement of N-terminal segments of differing length [73].

Despite the complexity of lipid vesicle binding, consensus was reached about the preferential interaction of αS with anionic phospholipids, although non-electrostatic interactions with neutral and zwitterionic lipids were also observed. A systematic study performed with phospholipid bilayer nanodiscs, an alternative model of lipid membranes exhibiting planar surfaces, confirmed the electrostatic model and showed that the binding mode was dependent on the relative abundance of anionic lipids versus neutral molecules [74]. Additionally, the degree of saturation and the length of the acyl chains were found to influence the binding of αS [75,76]. More specifically, molecular properties that determine the lipid phase state and membrane fluidity critically influence αS adsorption [74]. Interestingly, αS and anionic phospholipids may also form nanometer-sized lipoprotein particles, reminiscent of high-density lipoproteins, in which αS adopts a helical secondary structure [77]. Thus, the specific lipid environment has a profound impact on the partitioning and conformational transitions of αS, suggesting the possibility to tune molecular properties and regulate biomolecular and nano-bio interactions by careful design of the lipid-based nanomaterials.

### 2.4. Mixed-Type and Other Nanoparticles

The widely documented attraction of synuclein to lipid layers has inspired the development of lipid-based composite particles, other than simple vesicles. For example, Lee and coworkers used osmotic shock to coat monodisperse SNPs (60 nm diameter) with a lipid membrane, thereby obtaining spherical NP-supported lipid bilayers (SSLBs) [78]. Specifically, SNPs were amine-functionalized and the lipid coating was made by a mixture of the anionic lipid DOPA (1,2-dioleoyl-sn-glycero-3-phosphate) and the zwitterionic lipid DOPC (1,2-dioleoyl-sn-glycero-3-phosphocholine). SSLBs offer important advantages over SUVs/LUVs as they display larger X-ray scattering cross section of the silica core relative to membranes, enabling small-angle X-ray scattering (SAXS) and state-of-the-art X-ray photon correlation spectroscopy (XPCS), thereby expanding the repertoire of experimental techniques available to probe colloidal structure and dynamics of αS-bound vesicles. The authors reported that αS disrupted vesicle-vesicle interactions, with implications for synaptic membrane fusion and the ultrastructure and dynamics of synaptic vesicle pools.

The coating of inorganic NPs with lipid layers was also recently pursued by other groups [79]. The surface of AuNPs of varying size was capped with an inner layer of dodecanethiol and an outer layer of SDS. Thus, the organic bilayer was formed on a rigid scaffold and was resistant to deformation by αS, as opposed to lipid vesicles which are known to undergo structural remodeling upon interaction with the protein. By removing the effect of NP deformation, the obtained mimics allowed investigating the effects of NP curvature on protein binding behavior. SDS-AuNP—bound αS displayed increased solvent accessibility in the NAC region, suggesting that the adsorbed protein could possess higher aggregation propensity than unbound αS.

In the study of potential applications of NPs as protein aggregation modulators, Hajipour and coworkers focused on the interaction between αS and graphene sheets and superparamagnetic iron oxide NPs (SPIONs) with different surface properties and sizes [44]. Graphene sheets were prepared as small (150–250 nm), medium (450–650 nm) and large (800–1200 nm) sheets with polyglycerolsulfate, polyglycerol and polyglycerolamine coverages, displaying surface charges of −30 mV, 0 and +30 mV, respectively. The investigated SPIONs were 20, 50 and 100 nm large and displayed various functionalities (COOH, NH_2_, PEG-300 and chitosan). Atomistic MD simulations indicated that the interaction of αS with charged nano-objects was initially driven by electrostatic attraction, with a later involvement of hydrophobic residues and non-polar contacts dominating the interaction. Additionally, hydrogen bonds were formed, particularly in the case of amine-functionalized graphene. Differences in αS binding behavior to graphene and SPIONs were attributed to the distinct shape and corresponding surface curvature of the two materials. The affinity to neutral nano-objects was lower compared to charged ones. The various binding contributions resulted in distinct orientations of αS on the surface of different particles, supporting the view that distinct nanomaterials could differently affect αS self-assembly at the nano-bio interface.

Given the application of human serum albumin (HSA) NPs as carriers for drug delivery into the brain, HSA NPs were studied by Otzen and coworkers in order to assess the nature of their interaction with αS [80]. HSA NPs were produced as unmodified or polyethyleneimine(PEI)-functionalized ~35–40 nm spherical particles. By use of a centrifugation assay, fluorescence anisotropy measurements and small-angle X-ray scattering (SAXS) data, the authors found that αS was attracted much more strongly to positively charged PEI-HSA NPs than to negatively charged HSA NPs. Changes in the CD spectral shape suggested that the interaction with PEI-HSA NPs caused a conformational change in αS. The absence of a significant binding to HSA NPs, despite their net negative surface charge, may suggest that interactions with biological NPs are distinct relative to most synthetic platforms because the former exhibit an inhomogeneous distribution of polar, charged and nonpolar groups as opposed to the generally isotropic presence of chemical groups on the surface of common synthetized NPs.

## 3. Influence of Nanoparticles on Alpha-Synuclein Aggregate Formation

### 3.1. General Surface Effects on Aggregation Kinetics

**Figure 3:**
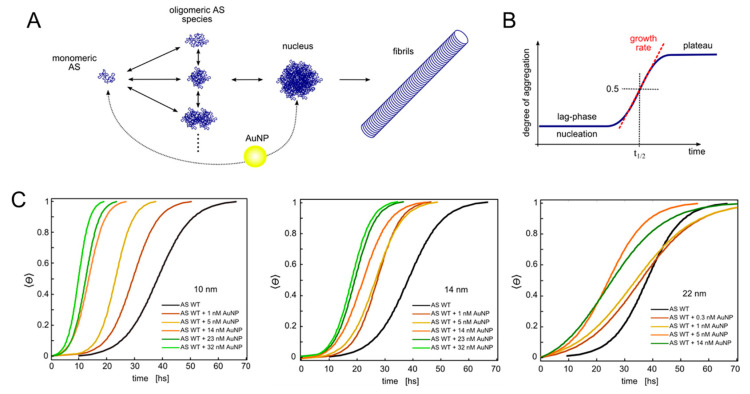
Nanoparticle-induced perturbation of fibril formation kinetics. (**A**) Schematic of the aggregation process of αS. AuNPs may interact with monomeric and/or oligomeric αS during the process of nucleation. (**B**) Diagram of the sigmoidal kinetics corresponding to nucleation followed by fast, autocatalytic growth. (**C**) Average θ(t) for samples with different concentrations of AuNPs with diameters of 10, 14 and 22 nm, respectively. θ(t) is the aggregate-responsive spectroscopic observable. Adapted with permission from Álvarez et al. Nano Lett, 2013, 13 (12) 6156–6163. Copyright 2013 American Chemical Society.

αS fibrillation is commonly considered a nucleation-dependent growth process that follows sigmoidal kinetics with distinct phases of nucleation, propagation and equilibration [81,82] (Figure 3A,B). The primary nucleation step (commonly associated with the lag-phase), during which soluble species are sequestered into oligomers of different sizes and structures, is much slower compared to the addition of monomers to preformed protofibrils, which leads to an exponential growth of fibrils (growth, propagation or elongation phase) until a state of equilibrium is achieved (plateau phase). This simplified two-step model adequately describes the evolution of the system from a macroscopic point of view, however it does not consider the multitude of microscopic events that contribute to the entire process. It is now established that simultaneous microscopic processes are ongoing during all phases and that processes other than primary nucleation and growth, such as fibril fragmentation or secondary nucleation, may represent important events during aggregation [83,84,85]. On-pathway and off-pathway intermediate supramolecular assemblies exhibit different degrees of cytotoxicity, however their identification and characterization remains challenging [81,86]. Due to the complexity of the aggregation phenomenon and the difficulty to access microscopic events, most studies involving perturbations of αS aggregation focus on comparative analyses of the kinetics at the macroscopic level. Experimentally, kinetics curves are generated by following time-dependent changes in sample turbidity or the fluorescence intensity of fibril-responsive dyes, such as Thioflavin T or by means of several other biochemical and biophysical techniques [21].

Surfaces have a profound impact on aggregation phenomena [42,87]. It has long been recognized that lipid membrane surfaces can act as catalysts of fibril formation, serving as a platform for nucleation and further polymerization [88,89]. Actually, membrane binding of αS appears to be an important factor in the pathogenesis of Parkinson’s disease [90]. Membrane-assisted aggregation could result from several concurrent factors, including a reduction of conformational entropy in the bound state, the induction of structural ordering, surface molecular crowding effects and lowering of the local dielectric constant which may facilitate the formation of intermolecular contacts [91,92,93]. The notion that surfaces may facilitate fibril formation is frequently exploited in in vitro aggregation studies of certain IDPs, including αS, which display extremely slow aggregation kinetics in solution. The addition of beads or other surfaces imparts a dramatic acceleration on protein self-assembly, allowing to perform experiments in practical time frames. Fibril seeds themselves offer particularly active surfaces that catalyze nucleation of aggregation-resistant proteins. Based on these evidences, NPs have attracted much interest owing to their high surface-to-volume ratio [94], compared to bulk material and many studies have been carried out on the effects of NPs on the aggregation of amyloidogenic proteins [22]. It emerges that NPs can lead to either acceleration or retardation of the fibril formation, depending on several factors such as the physicochemical properties of the NPs (e.g., size, charge, nature of exposed chemical groups), the amino acid composition and stability of the IDP, the concentrations of the solutes and the characteristics of the solution (e.g., ionic strength, pH) [95]. Thus, proper tuning of the NP properties shows promise as a means to control fibril formation pathways and could eventually provide a novel strategy for therapeutic intervention against aberrant protein aggregation in neurological disorders. Nonetheless, it must be considered that NP properties will also determine their ability to cross the blood-brain barrier and the cellular membranes before reaching the neuronal cytosol. Available pathways and strategies adopted to deliver NPs across the blood-brain barrier have been recently reviewed [96] and significant progress has been made in understanding the factors that control cellular uptake of NPs [97,98]. Most of the case studies presented here aimed at probing specific surface effects on protein aggregation and often did not address the ability of NPs to cross biological barriers.

A growing number of NP materials have been tested for their capability to influence the fibril formation process of αS. They include metals (Au, Fe) [41,99,100], metal/metalloid oxides (CeO_2_, TiO_2_, ZnO, Fe_3_O_4_, SiO_2_) [37,38,44,101,102,103,104], carbon (graphene, fullerenol) [44,105,106], polymers (dendrimers, others) [42,107,108,109,110,111], biomolecules (protein-based) [80,112] and lipids (detergent, phospholipid) [74,113,114,115]. The diversity of sizes, surface groups, charge density, hydrophobicity/hydrophilicity and other features of the investigated particles, render the rationalization of the results a challenging task. For example, both negatively and positively charged NPs were reported to inhibit αS fibrillation and both types were found to accelerate the process. In this regard, it appears that in order to establish more general paradigms, studies on a single protein/NP pair are of limited use, while more systematic investigations would be more informative. Furthermore, we emphasize the importance of conducting kinetics experiments with extreme rigor (e.g., avoiding NP precipitation, performing several replicas) and of carefully reporting the conditions in which experiments were performed (solution conditions, solute concentrations, mechanical agitation, etc.), to ensure that results can be compared and interpreted correctly.

A systematic study of the interaction between αS and citrate-capped AuNPs in a range of sizes (10, 14 and 22 nm) and of concentrations was carried out by Stefani and coworkers [41]. To monitor the evolution of the aggregation process at a macroscopic level, the team adopted an approach based on the use of the environment-sensitive fluorescent probe MFC, covalently attached to an engineered αS. This dual-emission dye is a multiparametric fluorescent probe, highly sensitive to changes in polarity and hence an exquisite reporter of early protein aggregation events [116]. The aggregation kinetics of αS in the presence of NPs followed a sigmoidal trend, exhibiting a transition halftime that decreased with the increase of AuNP concentration (Figure 3C). The overall acceleration produced by the AuNPs could be traced back to distinct effects on the nucleation and growth phases. For smaller AuNPs, the growth rate increased with particle concentration, while such effect was not observed with the 22 nm AuNPs. Instead, the latter had a more pronounced effect on the duration of the lag phase. The observed effects on the transition halftime and the growth rate did not scale with the available surface area. The authors attributed the reduction of the lag phase to the accumulation of protein on the AuNP surface, promoting the formation of critical nuclei for fibrillization. They further explained the variations in growth rate as a function of AuNP size by the formation of nuclei of different nature. This study therefore illustrates that the impact of NPs on the aggregation of αS is often not linear with some property of the NPs but multifaceted, underscoring the complexity of microscopic interactions occurring at the nano-bio interface.

### 3.2. Mechanistic Insights into Nanoparticle-Mediated Perturbations of αS Aggregation

A mechanistic description of the effects of surfaces on the aggregation kinetics of polypeptides requires consideration of the concurring microscopic events. In this respect, several available strategies may prove useful, such as the global analysis of macroscopic aggregation curves measured under different conditions [117], computational methods that take into account the structural plasticity of the polypeptide as well as the nature of the NP surface [117,118] and experimental procedures that allow the observation of structural transitions or the detection of transient intermediates. To date, a limited number of such studies have been applied to αS/NP systems.

The consideration that certain NPs accelerate protein aggregation, while others cause a retardation of fibril formation, prompted Linse and coworkers to explore which factors were responsible for one or the other behavior [42]. By use of a dynamic Monte Carlo method, the group simulated amyloid growth profiles in the presence of surfaces with varying attraction potential. The results showed that weakly attractive surfaces (peptide binding constant, K = 0.0017 μM^−1^) determined a reduction of the nucleation rate, compared to non-attractive surfaces, while highly attractive surfaces (K = 0.16 μM^−1^) accelerated the nucleation process. Indeed, weakly attractive objects simply reduce the concentration of free monomers available to form aggregation nuclei in solution but they do not change the microscopic events. By contrast, on the surface of highly attractive materials, distinct nuclei are formed in addition to those that form in solution. In the case of an intermediate attraction potential, the apparent kinetics was similar to that occurring with non-attractive surfaces, however the structures of the initial aggregates were different. Interestingly, the above observations were modulated by the intrinsic properties of the polypeptide, whereby for example weakly attractive surfaces retarded fibril formation by aggregation-prone mutants but accelerated the process for more stable polypeptides. ThT fluorescence assays performed using plain polystyrene NPs and αS consistently showed an acceleration of fibril growth upon increasing NP concentration, as a consequence of a strong non-electrostatic attraction causing surface-catalyzed nucleation. In general, it seems possible that positively and negatively charged NPs could exert the same inhibitory or acceleratory effect on the fibrillization of αS, because they would establish electrostatic interactions with either the acidic C-terminal or the basic N-terminal domains [56]. Neutral objects were reported to have limited impact on the aggregation rate, presumably because they were not able to interact significantly with αS [44].

While computational methods can capture the formation of early-stage aggregates and provide atomistic insights into conformational transitions with relative ease, the identification of transient intermediates and the detection of time-dependent structural changes during fibrillization remain experimentally challenging. Chattopadhyay and coworkers applied fluorescence correlation spectroscopy (FCS) and laser scanning microscopy (LSM) to investigate the early events of αS aggregation in the presence of pristine or Lys-modified Fe_3_O_4_ NPs [43]. The use of these techniques proved advantageous over the use of standard dye-based methods which are quite insensitive to the formation of smaller aggregation intermediates. Bare Fe_3_O_4_ NPs were found to accelerate early and late-stage aggregation of αS, while Lys-coated Fe_3_O_4_ NPs displayed an inhibitory effect. Maximum entropy analysis of the correlation functions measured by FCS detected increases in conformational heterogeneity during the progress of aggregation and revealed the presence of aggregated species at earlier time points in the presence of bare Fe_3_O_4_ NPs. The early aggregates were visualized by LSM. The molecular basis for the different perturbations elicited by bare and Lys-coated particles remains elusive, however the study demonstrated the possibility of acquiring important information on aggregates in heterogeneous systems.

Hajipour and colleagues used size exclusion chromatography coupled with multi-angle light scattering (SEC-MALS) to quantify the amount of small αS oligomers formed in the presence of SPIONs or graphene nanoobjects and ranked the particles according to their ability to trigger the formation of such aggregates [44]. They were further able to determine whether the formed oligomers were on- or off-pathway with respect to fibrillization. In another study, Tira et al. followed the time course of αS aggregation in the presence of SNPs using CD spectroscopy [38]. The authors could track the time-dependent structural transitions of the polypeptide from its native disordered state to conformations with mixed α and β secondary elements. Interestingly, the shapes of CD spectra collected at different time points for αS and its methionine-oxidized form in the presence of SNPs indicated that the two species formed distinct supramolecular assemblies, consistent with the reported resistance of the oxidized species to form fibrils [119]. Taken together, the information obtained from experiments on prefibrillar species could be used to inform computational methods and contribute to an improved description of the complex aggregation pathways.

### 3.3. Lipid Surface-Mediated αS Aggregation

The nature of lipid surfaces is quite unique due to their soft, dynamic character which allows peptides to penetrate into the lipid layer(s), at least partially [120,121]. In addition, lipids and amyloidogenic polypeptides share an amphipathic structure and are therefore intrinsically prone to interact. The interactions are modulated by several factors and are highly dependent on lipid composition, surface charges and thermotropic properties [122,123]. Hence, the mechanisms by which lipid nanovesicles affect αS fibril formation may depart from those involved with other nanomaterials. Indeed, the mode of association with lipid surfaces modulates αS aggregation in different ways [91,115,124,125]. Furthermore, it has been suggested that fibrillization in the presence of lipid molecules may result in the formation of protein-lipid co-aggregates [126,127,128] and that the morphology of fibrils is modulated by the relative proportion of protein and lipids [129].

αS partitions dynamically to SUVs and LUVs composed of anionic phospholipids such as phosphatidylserines [39,123]. Interestingly, the binding affinity is highest when the lipid layers are in the fluid state, as opposed to the gel-like state, since in the former case the hydrophobic portions of the lipid molecules are on average more exposed [123]. In turn, αS binding affects the lipid phase behavior and induces lipid segregation into protein-poor and protein-rich populations [123]. Yet, unexpectedly, the lipid phase state does not correlate with the vesicle-promoted acceleration of αS amyloid fibril formation [123]. Instead, the kinetics of aggregation was found to correlate with the solubility of the lipid molecules, suggesting that at least part of the free energy barrier for the aggregation process is associated with the translocation of lipid molecules from a membrane to a protein environment [123].

Aggregation assays carried out under quiescent conditions and at varying dimyristoyl phosphatidylserine (DMPS)/αS ratios indicated that αS did not convert into fibrils when excess lipid was present and most of the protein was in the bound state [39]. On the contrary, at lower lipid concentrations, when significant populations of both free and lipid-bound protein were established, SUVs determined the rapid formation of amyloid fibrils. Indeed, a combined experimental and theoretical analysis indicated that at low lipid/αS ratios, the bound protein promoted a primary nucleation process, much faster than that occurring in bulk solution. The facilitated nucleation was attributed to the high local concentration of protein on the SUV surface and to a conformational shift towards aggregation-competent states [39]. Under the used conditions, other microscopic processes, including homogeneous primary nucleation, secondary nucleation and fragmentation, did not contribute measurably to the aggregation reaction. A similar finding was reported concerning the effect of nanovesicles made by zwitterionic lipids, which influenced the lag time more than the fibril elongation rate [113]. Under quiescent conditions, secondary processes may be prevented due to kinetic trapping of the lipid-bound fibrils [39]. Site-resolved NMR data obtained using nanodiscs as membrane models indicated that region-specific membrane affinities (particularly of the NAC region) were correlated with aggregation behavior [74]. The dual effect of lipid surfaces to both accelerate or inhibit αS amyloid fibril formation depending on the relative proportion of protein and phospholipids may suggest a possible mechanism for the onset of aberrant aggregation as a consequence of altered levels of αS expression, associated with some forms of PD [130].

It has been proposed that the transient interaction of αS with lipid bilayers may determine the formation of a pool of helical conformers that are aggregation-resistant [131]. Thus, physiologically, lipid surfaces may act as chaperones that assist the folding process of otherwise disordered protein molecules. An imbalance in the relative populations of protein in the folded and unfolded pools may cause aberrant aggregation in pathology. These findings suggest the possibility to develop tailored NPs as artificial cofactors that assist the formation of aggregation-resistant αS species.

### 3.4. Nanoparticle-Fibril Interactions and Fibril Disassembly

The removal of amyloid deposits is a prominent therapeutic aim in protein misfolding diseases [132]. However, the disassembly of preformed amyloid fibrils is both challenging and risky. On the one hand, protein fibrils are insoluble, extremely stable and resistant to degradation. On the other hand, disaggregation may exacerbate amyloid toxicity by increasing the load of toxic oligomers [133]. Thus, in order for NPs to be effective in reverting aberrant deposition of protein fibrils, they must display significant affinity for the fibrillar structures, establish interactions that weaken or disrupt the dense network of hydrogen bonds that stabilize the stacked β-strands and promote the conversion of fragments into harmless products. Diverse NPs were shown to interact with preformed αS fibrils.

Citrate-capped AuNPs of the size of 22 nm but not of 14 nm and lower, were found to associate with fibrils, indicating a size-dependent affinity [41]. Mercapto-undecanesulfonate-coated AuNPs were developed to target synthetic, recombinant and native fibrils derived from different amyloidogenic proteins, including αS [134]. Such particles did not exhibit fibril-disaggregating properties, instead they were exploited to label amyloid fibrils for assessing morphological polymorphism using cryogenic electron microscopy (cryo-EM) [134]. Both SPIONs and graphene were reported to disassemble αS fibrils, with a higher efficacy shown by positively charged nano-objects, possibly related to their higher affinity to charged αS residues [44]. Upon fibril fragmentation, the amount of oligomeric species did not increase, suggesting a safe use of these NPs [44].

In addition to graphene, also graphene quantum dots (GQDs) were shown to induce dissociation of αS fibrils [40]. Ko and coworkers carried out a thorough characterization of this process [40]. GQDs produced the dissociation of fibrils into short fragments of an average length of 235 nm and 70 nm after 6 and 24 h, respectively. After 3 days of incubation, the number of fragments decreased, indicating that the process progressed until complete disassembly. The interaction of the negatively charged GQDs with αS was likely initiated by electrostatic attraction with the protein’s N-terminal domain. MD simulations performed with the sole NAC domain indicated that after initial binding, the β-sheet structure of the outer monomer was rapidly and completely destroyed as a consequence of strong hydrophobic interactions between GQDs and valine residues. Importantly, GQDs could penetrate the blood-brain barrier and protect mice against dopamine neuron loss induced by preformed amyloid deposits.

Dendrimers have been recognized as potential powerful agents for the disaggregation of fibrils, displaying strong binding affinity to fibrillar structures and exerting their destructive effect by acting as efficient chaotropes [135]. Specifically, polyamidoamine (PAMAM) dendrimers were shown to interact with αS fibrils, the binding affinity increasing with the generation number (G4 < G5 < G6) [109]. The combined evidence from TEM, CD and ThT fluorescence indicated that dendrimers attacked the fibrils along the entire filament, not just at the ends and lead to amorphous aggregation. Similar to PAMAM dendrimers, also urea(U)- and methylthiourea(MTU)-modified polypropyleneimine (PPI) dendrimers were found to disaggregate preformed fibrils [136]. TEM detected the fragmentation of fibrils by G3-MTU-PPI into smaller and less organized aggregates. Interestingly, both types of NPs were found to significantly reduce the αS fibril load inside SK-MEL-5 cells in a dose-dependent and generation-dependent (MTU-PPI) or generation-independent (U-PPI) manner. However, MTU-PPI dendrimers displayed higher cytotoxicity in the presence of preformed αS fibrils [136]. Further studies on the effects of dendrimers and other polymeric particles on αS aggregation have been reviewed elsewhere [137].

## 4. Alpha-Synuclein/Nanoparticle Conjugates and Hybrid Nanomaterials

Functionalization or the combination of NPs with αS molecules has been intensely explored to produce nanobioconjugates and hybrid nanomaterials for diverse technological applications in bioanalytical chemistry and bionanotechnology [45,138,139]. Depending on the NP material and the aim of the application, a variety of conjugation strategies have been exploited, ranging from the simple deposition of the biomolecule on the NP surface [46,139], to non-covalent high affinity binding [45] and to covalent bond formation [138,140]. The conformational versatility of αS made it possible to exploit different properties of the associated molecules, from a disordered and highly dynamic form to the ordered superstructure typical of fibrils. Furthermore, protein self-assembly was exploited to fabricate ordered multi-component, supramolecular nanomaterials [46].

Jares-Erijman and coworkers explored the possibility to develop novel reactive agents or nanoactuators, based on the decoration of QDs with multiple copies of αS [45]. αS containing an A90C single point mutation was conjugated to biotin via maleimide chemistry, while QDs were capped with streptavidin. Thus, αS-QD nanoconjugates were obtained exploiting the formation of the well-known high affinity biotin-streptavidin complex. The nanoactuators were found to accelerate the formation of αS amyloid fibrils, both in vitro and in live cells, thereby acting as artificial nucleation seeds. The catalytic effect was attributed to the self-assembly of the protein initiated by the high local concentrations displayed at the surface of the NPs. The developed system could facilitate cellular studies of amyloid formation.

The production of AuNP-biomolecule conjugates often exploits the spontaneous formation of ligand monolayers via unique bonds between the Au surface and sulfhydryl groups of the biomolecule. Paik and coworkers found that a thin shell of modified αS molecules conjugated to AuNPs via Au-S bonds facilitated their deposition into a regular two-dimensional array on a glass support [138]. The obtained material constituted the basis for the production of a surface-enhanced Raman scattering biosensor. The platform was indeed responsive to phthalocyanine tetrasulfonate, an αS ligand and aggregation inhibitor and to metal ions forming complexes with the compound. The formation of a uniform array of coated AuNPs was attributed to the capability of fixed αS molecules to establish intermolecular interactions.

**Figure 4:**
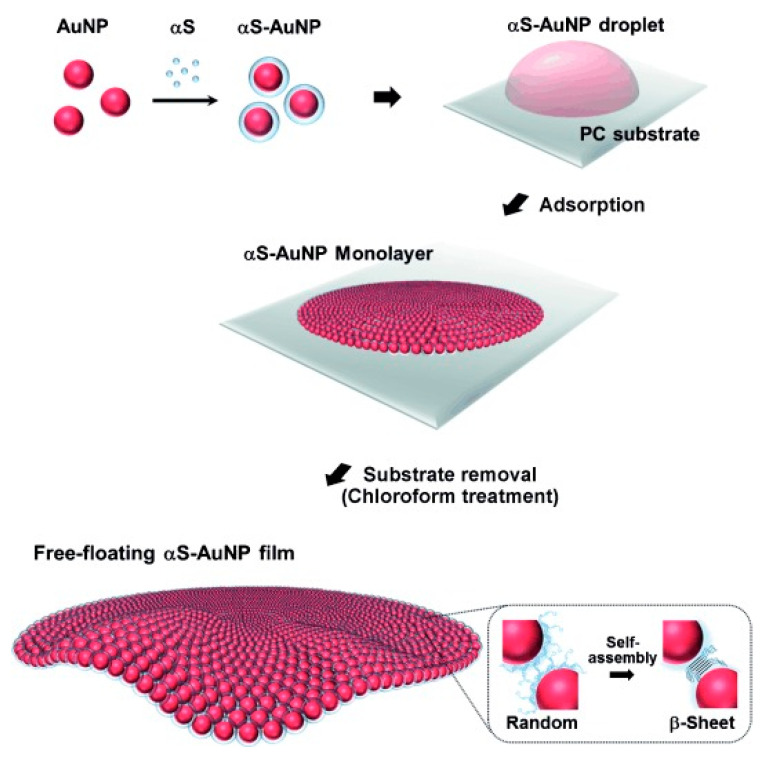
Production of functional αS-based nanobiocomposites. The fabrication of a free-floating αS-AuNP monolayer film through the preparation of αS-coated AuNPs, adsorption of the αS-AuNPs onto the polycarbonate (PC) substrate and subsequent free-standing film production by αS–αS self-assembly upon removal of the substrate with chloroform. Reproduced with permission from Lee et al. Angew Chem. Int. Ed., 2015, 54 (15), 4571–4576, © 2020 WILEY-VCH Verlag GmbH & Co. KGaA, Weinheim.

The αS-mediated assembly of AuNPs into hierarchical superstructures was further exploited to fabricate a flexible, free-standing NP monolayer film useful for the development of bio-integrated nano-devices and high-performance sensors [46] (Figure 4). Cysteine-free protein was first adsorbed onto AuNPs (10–30 nm) and subsequently onto a polycarbonate substrate. Fibril-like protein-protein linkages were revealed by scanning electron microscopy and β-sheet structure formation was observed by attenuated total reflectance Fourier transform infrared spectroscopy. The conversion into β structures was probably triggered by the exposure of the protein-AuNP system to chloroform, necessary to release the film from the support. Importantly, the process was specific for αS, as the film could not be obtained with other model protein molecules. An analogous concept was used to develop metal NP-based organic field-effect transistors with electrical memory [139]. A closely packed AuNP monolayer was obtained by the pH-dependent adsorption of αS-AuNP conjugates on a SiO_2_ surface. Pentacene, a high-performance organic semiconductor, was finally deposited on the formed αS-AuNP film. The use of αS proved invaluable for the optimal controllability over the hybrid material structures, allowing to obtain highly tunable memory performance.

Another study demonstrated the possibility to produce αS-based nanocomposites as intracellular drug delivery systems [140]. Mesoporous SNPs (~100 nm size and pore diameter of 2–3 nm) were coated with AuNPs, previously functionalized with αS, to yield ‘raspberry-type’ particles-on-a-particle (PoP) structures. The anticancer agent rhodamine 6G, loaded into the PoPs, could be released upon exposure of the nanocomposite to the αS-binding cation Ca^2+^. Intracellular uptake of PoPs and drug release was demonstrated with HeLa cells in the presence of intracellular Ca^2+^-regulating agents. In this context, αS acted as a useful switch to open the gates of mesoporous SNPs by altering its conformation in a Ca^2+^-dependent manner.

Assembly of NPs into controllable nanoobjects expands their application potential for the development of nanoscale electronic and optical devices. Biopolymers have been shown to provide effective means for organizing NPs into superstructures and fibrillar structures have attracted considerable interest in this respect. For example, pea-pod-type chains of AuNPs embedded into dielectric αS fibrils were shown to exhibit photoconductivity with visible light [141]. The unit assembly strategy of amyloid fibril formation of αS was employed to construct anisotropic one-dimensional chains of AuNPs within the amyloid fibrils. The necessary conformational transition of AuNP-adsorbed molecules from the disordered to the ordered state was induced by exposure of the assembly units to hexane or pH change. Interestingly, the morphological polymorphism of fibrillar structures obtained in different conditions could provide a means to fabricate chains of AuNPs with diverse organization. NP chains of Pd and Cu were also synthetized exploiting αS fibrils as biopolymeric templates [142]. Finally, αS amyloid fibrils were found to drive a helical arrangement of gold nanorods [143]. The latter showed no apparent interaction with the monomeric protein but effective adsorption onto chiral fibril structures via noncovalent interactions. The helical arrangement resulted in intense optical activity at the surface plasmon resonance wavelengths, thereby constituting a novel sensing technique for the detection of fibrils.

## 5. Conclusions

We surveyed research works focused on the behavior of the small protein αS at the interface with NPs in an attempt to obtain a more defined picture of the factors that determine the protein-particle interactions and influence the conformational transitions of the biomolecule. Through a better understanding of αS-NP systems at the molecular level, we have moved forward towards the possibility of designing nanomaterials with useful functionalities for applications in diverse scientific and technological areas. A number of studies have demonstrated that it is possible to determine and even predict how αS interacts with different nanoscale surfaces, highlighting the multiple conformations that the polypeptide can adopt and describing how to control the interactions. It has emerged that electrostatic forces dictate the mode of adsorption of monomeric αS to diverse nanoscale surfaces, providing alternative anchors (the amphiphilic N-terminal or acidic C-terminal domains) for binding to negatively or positively charged NPs. Yet, hydrophobic attraction was shown to contribute to protein adsorption too, determining some involvement of the aggregation-promoting NAC domain. It has been shown that certain NPs are able to modify protein aggregation, with encouraging results regarding the possibility of redirecting the formation of neurotoxic aggregates towards more harmless species or even of disassembling otherwise intractable amyloid fibrils. The different exposure of amino acid residues in the monomeric, oligomeric and fibrillar states of αS may provide the basis for the selective targeting of toxic species by NPs. Finally, the extraordinary conformational plasticity of αS was exploited to design higher hierarchical structures that showed interesting novel physical or chemical attributes. The field of study continues to show extraordinary vivacity and it is expected that new therapeutic and technological solutions will be proposed in the near future.
